# Efficacy and Safety of Pyrotinib Versus T-DM1 in HER2+ Metastatic Breast Cancer Patients Pre-Treated With Trastuzumab and a Taxane: A Bayesian Network Meta-Analysis

**DOI:** 10.3389/fonc.2021.608781

**Published:** 2021-05-03

**Authors:** Hao Liao, Wenfa Huang, Yaxin Liu, Wendi Pei, Huiping Li

**Affiliations:** ^1^ Key Laboratory of Carcinogenesis and Translational Research (Ministry of Education/Beijing), Department of Breast Oncology, Peking University Cancer Hospital and Institute, Beijing, China; ^2^ Department of Hematology-Oncology, International Cancer Center, Shenzhen University General Hospital, Shenzhen University Health Science Center, Shenzhen, China; ^3^ Center for Reproductive Medicine, Department of Obstetrics and Gynecology, Beijing Key Laboratory of Reproductive Endocrinology and Assisted Reproductive Technology and Key Laboratory of Assisted Reproduction, Ministry of Education, Peking University Third Hospital, Beijing, China

**Keywords:** network meta-analysis, trastuzumab emtansine, pyrotinib, human epidermal growth factor, metastatic breast cancer

## Abstract

**Purpose:**

To compare the efficacy and safety between pyrotinib (Pyr) and trastuzumab emtansine (T-DM1) in pre-treated human epidermal growth factor receptor 2-positive (HER2+) metastatic breast cancer (MBC) patients.

**Methods:**

A comprehensive literature search of the PubMed, EMBASE, and Web of Science was performed in August 2020. Randomized clinical trials comparing the efficacy and safety between different anti-HER2 regimens in patients pre-treated with trastuzumab (Tra) and a taxane in metastatic settings (≤second-line treatment) were included. A fixed effects network meta-analysis based on the Bayesian inferential framework was conducted for progression-free survival (PFS), overall survival (OS), overall response rate (ORR), and grade ≥3 adverse events (AEs). Values of surface under cumulative ranking probability curve (SUCRA) were calculated to offer a ranking of all regimens.

**Results:**

Twelve studies with 4,353 subjects were identified. Nine regimens were included into the network: T-DM1, lapatinib-capecitabine (Lap-Cap), Tra-Cap, Cap, neratinib (Ner), pertuzumab (Per)-Tra-Cap, Pyr-Cap, atezolizumab (Ate)-T-DM1, and Ner-Cap. For PFS, Pyr-Cap was more favorable than T-DM1 (hazard ratio, 95% confidence interval: 0.77, 0.70–0.86), Lap-Cap (0.64, 0.59–0.69), Tra-Cap (0.63, 0.56–0.70), Cap (0.50, 0.45–0.56), Ner (0.59, 0.51–0.69), Per-Tra-Cap (0.68, 0.59–0.79), and Ner-Cap (0.72, 0.64–0.81). For OS, Pyr-Cap showed further improvement than Lap-Cap (hazard ratio, 95% confidence interval: 0.71, 0.52–0.99), Cap (0.68, 0.49–0.96), and Ner (0.65, 0.45–0.94). For ORR, Pyr-Cap was significantly superior than Cap (odds ratio, 95% confidence interval: 7.87, 1.22–56.51). No significant difference was observed in grade ≥3 AEs among all the regimens. Pyr-Cap ranked in the highest in PFS, OS, ORR, and grade ≥3 AEs (SUCRA = 99.4, 89.7, 86.4, and 89.3%).

**Conclusions:**

These results indicate that Pyr may be more effective than T-DM1 in HER2+ MBC patients pre-treated with Tra and a taxane. However, it may be associated with more grade ≥3 AEs.

## Introduction

Breast cancer (BC), a highly heterogeneous disease, currently represents the most common cancer in females with over million new cases confirmed per year worldwide (1, 2). According to the molecular landscape of the tumor, BC is divided into four subtypes including luminal A, luminal B, human epidermal growth factor receptor 2-positive (HER2+), and triple-negative BC (3). The HER2 is a receptor tyrosine-protein kinase and amplified in 15–30% of all human BC (4). Positive HER2 is a risk factor for BC patients, which is reflected in the aggressive clinical phenotype and poor prognosis for HER2+ BC patients (5). The first FDA-approved HER2-targeted agent, trastuzumab (Tra; Herceptin™, Roche), started the new era of targeted therapy of BC. The administration of Tra either in combination with chemotherapy or in monotherapy has significantly improved the disease-free and overall survival (OS) of patients in the neo/adjuvant treatment phase, as well as in the rescue treatment for advanced/metastatic BC (MBC) (6–8). In addition to Tra, numerous novel agents including trastuzumab emtansine (T-DM1; Kadcyla™, Roche), pertuzumab (Per; Perjeta™, Roche), lapatinib (Lap; Tyverb™, GlaxoSmithKline), neratinib (Ner; Nerlynx™, Puma Biotechnology), and pyrotinib (Pyr; HengRui) have been introduced in recent years (9–13). Despite all the advances in targeted therapy, the resistance to anti-HER2 treatment remains a major challenge (14). On the other hand, there are controversies in choosing which anti-HER2 regimen for HER2+ patients who progressed on prior treatment of Tra and a taxane (15). Therefore, it is of necessity and interest to identify the potential best anti-HER2 regimen in later treatment lines of HER2+ MBC.

T-DM1 is a first-in-class antibody-drug conjugate consisting of Tra linked to an anti-tubulin agent DM1, *via* a stable thioether linker (16). T-DM1 has shown its efficacy both in early-stage and advanced-stage HER2+ BC (9, 17). The results of EMILIA phase III study (Clinical-Trials.gov number: NCT00829166) indicated that T-DM1 significantly prolonged the progression-free survival (PFS) (9.6 *vs.* 6.4 months; hazard ratio (HR), 95% confidence interval (CI): 0.65, (0.55–0.77)) and OS (29.9 *vs.* 25.9 months; HR, 95%CI: 0.75, (0.64–0.88)) of HER2+ MBC patients pre-treated with Tra and a taxane, with less toxicity compared with Lap plus capecitabine (Cap; Xeloda™, Roche) (9, 18). Based on these findings, T-DM1 was approved for the second-line treatment of HER2+ MBC in 2013 (19). Pyr, a novel irreversible pan-ERBB receptor tyrosine kinase inhibitor, has shown promising anti-tumor activity and acceptable tolerability in a phase I study (Clinical-Trials.gov number: NCT01937689) in 2017 (20, 21). In a phase II, randomized, multi-center, open-label study (Clinical-Trials.gov number: NCT02422199), Ma et al. further evaluated the efficacy and tolerability of Pyr plus Cap compared with Lap plus Cap in HER2+ MBC patients pre-treated with taxanes, anthracyclines, and/or Tra. For patients with prior anti-HER2 treatment, Pyr plus Cap yielded significantly longer PFS (HR, 95%CI: 0.37, (0.19–0.74), log-rank *P* = 0.0031) than Lap plus Cap (13). According to the results of phase I/II studies, Pyr was recommended to replace Tra for the second-line anti-HER2 therapy of HER2+ MBC patients in the 2019 CSCO (Chinese Society of Clinical Oncology) BC guidelines (13, 21).

Both T-DM1 and Pyr showed favorable therapeutic effects and safety in randomized controlled trials (RCTs), nevertheless, there is currently no head-to-head comparison between the two agents. Network meta-analysis is a highly attractive, new type of systematic review and meta-analysis (22). In the last decade, network meta-analysis has become increasingly popular, as it synthesizes direct and indirect evidence to compare multiple interventions in a network of RCTs, overcoming the limitation of traditional pare-wise meta-analysis (23–25). Therefore, we conducted this updated network meta-analysis based on a Bayesian inferential framework to evaluate the efficacy and safety of Pyr versus T-DM1 in HER2+ MBC patients pre-treated with Tra and a taxane.

## Methods

This network meta-analysis was conducted and reported according to the Preferred Reporting Items for Systematic Reviews and Meta-Analysis (PRISMA) statement (26).

### Search Strategy

Relevant articles were obtained by searching Medline/PubMed, EMBASE, and Web of Science databases through 15 August 2020. The detailed search strategy was as follows: (breast OR mammary) AND (cancer OR neoplasm OR oncology OR tumor OR malignancy OR carcinoma OR adenocarcinoma OR sarcoma) AND (metastasis OR metastatic OR advanced OR secondary OR recurrent OR inoperable OR unresectable OR disseminated OR incurable) AND (“human epidermal growth factor receptor 2” OR HER2 OR HER-2 OR Her-2 OR ERBB2 OR neu) AND (positive OR enriched OR overexpressing OR overexpressed) AND (trial OR study) AND (randomized OR randomized OR randomly OR randomization OR RCT) AND (trastuzumab OR Herceptin) AND (Anti-HER2 OR HER2-targeted OR lapatinib OR tykerb OR neratinib OR pyrotinib OR pertuzumab OR T-DM1 OR “trastuzumab emtansine” OR trastuzumab-DM1 OR trastuzumab-MCC-DM1 OR margetuximab OR tucatinib OR “trastuzumab deruxtecan” OR DS8201 OR poziotinib OR afatinib OR everolimus OR chemotherapy OR cyclophosphamide OR methotrexate OR fluorouracil OR 5FU OR 5-FU OR doxorubicin OR mitoxantrone OR epirubicin OR paclitaxel OR docetaxel OR liposomal doxorubicin OR nab-paclitaxel OR “nab paclitaxel” OR eribulin OR capecitabine OR vinorelbine OR carboplatin OR cisplatin OR platinum OR gemcitabine) (**Appendix 1**). Manual searches of conference abstracts from the American Society of Clinical Oncology (ASCO) and the European Society for Medical Oncology (ESMO) were conducted to find additional eligible studies.

### Selection of Studies

This network meta-analysis included studies that met the following criteria: 1) RCT of adults (≥18 years old); 2) HER2+ MBC patients confirmed by centralized testing (3+ [immunohistochemistry] or copy number amplification [fluorescence *in situ* hybridization]); 3) patients pre-treated with Tra and a taxane in metastatic settings (≤second-line treatment); 4) patients were treated with anti-HER2 regimens or combination of anti-HER2 regimens and chemotherapy in the intervention group, while the control group received other anti-HER2 regimens or chemotherapy alone; 5) measurements of PFS, OS, overall response rate (ORR), and grade ≥3 adverse events (AEs); 6) written in English. The main exclusion criteria were as follows: 1) repeated reports; 2) non-RCT studies; 3) non-English studies; 4) major defects in research design; 5) statistical methods were wrong and could not be corrected; 6) patients received first-line treatment; 7) patients were pre-treated with ≥ third-line anti-HER2 treatment. All searched articles were screened according to the titles and abstracts for exclusion. Candidate full-text studies were then assessed before final inclusion.

### Data Extraction and Risk of Bias Assessment

Two reviewers independently extracted the data from included studies by a pre-specified protocol. In this process, the following study characteristics were collected: first author, publication year, study design, sample size, current treatment strategy, previous anti-HER2 treatment, and main outcomes (PFS, OS, ORR, and grade ≥3 AEs).

The Cochrane Risk of Bias tool was used to assess the risk of bias of the included primary studies by the two reviewers independently (27). All included studies were evaluated as high, low, or unclear risk according to the documented methodological quality. Any disagreements between the two reviewers were resolved by discussion and team consensus.

### Statistical Methodology

Firstly, the evidence of network was generated in Stata 15.0 (StataCorp, College Station, TX, USA) using Network package. The width of edge corresponded to the number of studies comparing each pair of regimens and the size of node was proportional to the number of randomized participants. To compare the therapeutic effects and safety of different anti-HER2 regimens, log HR for time-to-event data (PFS and OS) and log odds ratio (OR) for binary variables (ORR and grade ≥3 AEs) of two regimens were calculated. For PFS, OS, and grade ≥3 AEs, the therapeutic effects of one regimen were better than the other one when the corresponding HR/OR value was less than 1. For ORR, in the case that the corresponding OR value was over 1, the therapeutic effects of one regimen surpassed the other one.

This network meta-analysis was conducted based on a Bayesian, fixed effects, consistency model *via* Markov Chain Monte Carlo modeling (28). We chose the Bayesian inferential framework because it allows external information to be included and can capture and propagate uncertainty. In addition, it will not be biased by small sample size, while the frequentist inference often becomes biased when the sample size decreases (29, 30). The OpenBUGS 3.2.3 (members of OpenBUGS Project Management Group, see www.openbugs.net) and GeMTC 0.14.3 (Generate Mixed Treatment Comparisons, see http://drugis.org/software/addis1/gemtc) were used for the analysis of time-to-event data (PFS and OS) and the analysis of binary variables (ORR and grade ≥3 AEs), respectively. The parameters in OpenBUGS and GeMTC were set as follows: initial value, 2.5; number of simulation iterations, 50,000; number of adaptations, 5,000; thinning factor, 10; and number of chains, 3. In order to accurately rank the treatment effects and safety of all regimens, values of surface under cumulative ranking probability curve (SUCRA) were calculated and cumulative probability curves were plotted in Stata 15.0 (25). The value of SUCRA would be 1 when a treatment is certain to be the best one or 0 when a treatment is certain to be the worst one (31).

The Deviance Information Criterion (DIC) was used to assess the model inconsistency for PFS and OS in OpenBUGS. The lower the DIC value, the lower the inconsistency of the model (32). For ORR and grade ≥3 AEs, the inconsistency was evaluated in GeMTC based on the value of inconsistency factor. If the data were consistent, the inconsistency factor would be expected to be close to 0 and its 95%CI would contain the neutral value (0). In addition, the random effects standard deviation in the consistency model would be expected to be roughly equal with that in the inconsistency model when the data were inconsistent (33). 

## Results

### Study Inclusion and Characteristics

**Figure 1:**
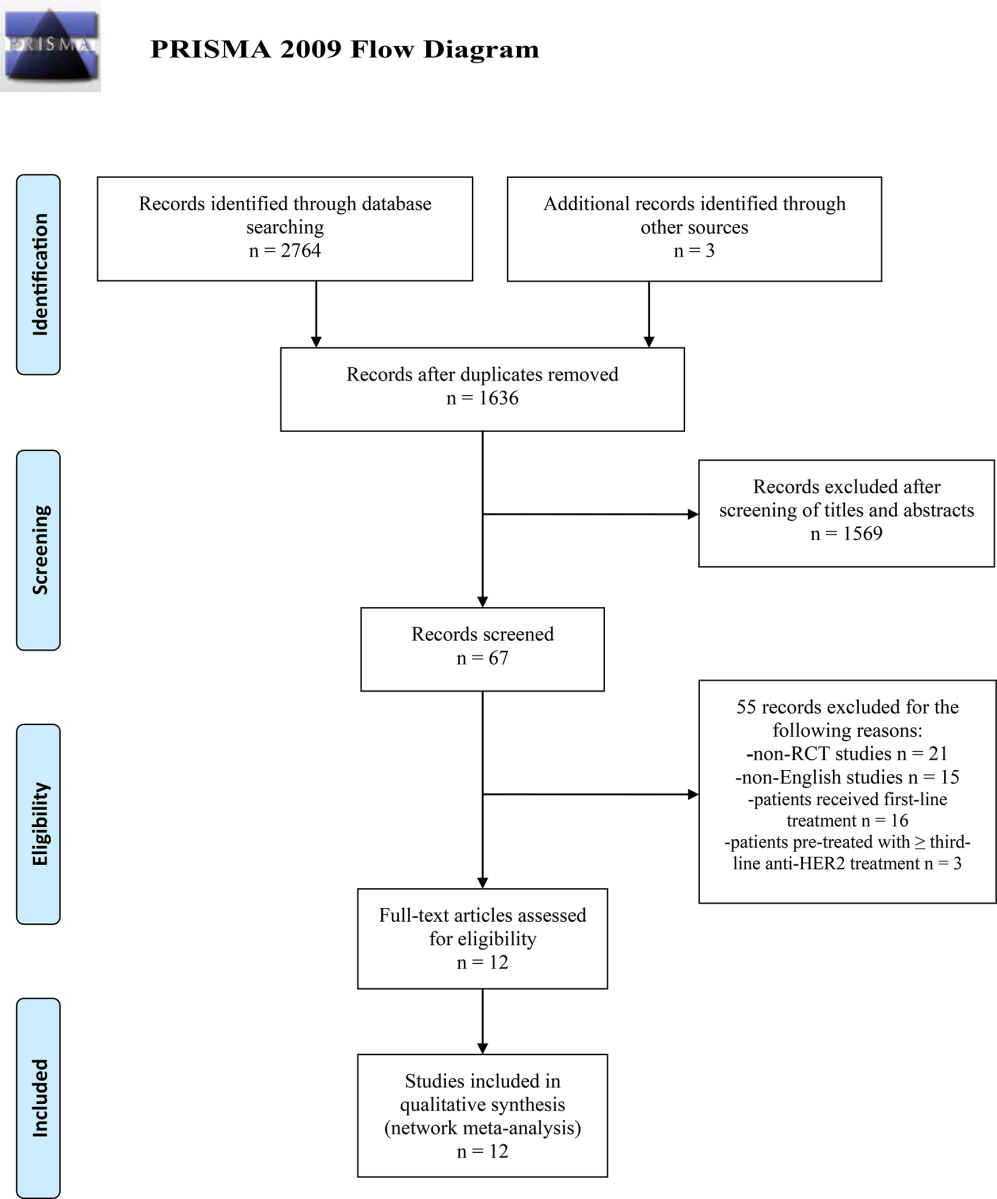
PRISMA 2009 Flow Diagram of study selection process. PRISMA, Preferred Reporting Items for Systematic Reviews and Meta-Analysis; RCT, randomized controlled trial; HER2, human epidermal growth factor receptor 2-positive.

The search was conducted and completed on August 15, 2020. The PRISMA 2009 Flow Diagram of study selection process was shown in **Figure 1**. A total of 2,764 records were initially retrieved through database search. Three records were obtained from the conference abstracts of ASCO and ESMO. Among them, 1,131 records were duplicates. Then, we excluded 1,569 irrelevant records after screening the titles and abstracts. After a further evaluation, 55 records were excluded: 21 non-RCT studies; 15 non-English studies; 16 studies of patients who only received first-line treatment; three studies of patients who were pre-treated with ≥ third-line anti-HER2 treatment. Finally, twelve studies were included for this network meta-analysis (8–13, 34–39). No additional studies were obtained through checking the reference lists of these articles. The basic characteristics of included studies were presented in **Table 1**. All included studies are multi-center RCTs published after 2000. Seven studies are phase III trials (9–12, 34, 37, 38), while the other five studies are phase II trials (8, 13, 35, 36, 39). The sample size of individual study ranges from 86 to 991, with a total of 4,353 subjects enrolled in these studies. All studies focus on the efficacy and safety of anti-HER2 regimens in HER2+ MBC patients who progressed on Tra and a taxane.

**Table 1:** Basic characteristics of included studies.

Author	Study design	Study phase	Population (n) treatment/control	Treatment	Control	Previous trastuzumab settings	Current treatment lines	Main outcomes^†^
Geyer et al. (34)	Multi-center RCT	III	198/201	Lap-Cap	Cap	Adju 17 meta 296	2L: 393	1, 2, 3, 4
von Minckwitz et al. (8)	Multi-center RCT	II	78/78	Tra-Cap	Cap	Adju 3 meta 153	1L: 3 2L: 153	1, 2, 3, 4
Verma et al. (9)	Multi-center RCT	III	495/496	T-DM1	Lap-Cap	Adju 155 meta 836	1L: 118 2L: 361 3L-: 512	1, 2, 3, 4
Martin et al. (35)	Multi-center RCT	II	117/116	Ner	Lap-Cap	Adju 60 meta 171	2L: 32 3L-: 200	1, 2, 3
Pivot et al. (11)	Multi-center RCT	III	271/269	Lap-Cap	Tra-Cap	Adju 151 meta 189	1L: 238 2L: 302	1, 2
Urruticoechea et al. (10)	Multi-center RCT	III	228/224	Per-Tra-Cap	Tra-Cap	Adju and meta 115 meta 334	2L: 449	1, 2, 3, 4
Takano et al. (36)	Multi-center RCT	II	43/43	Lap-Cap	Tra-Cap	Adju 5 meta 81	1L: 5 2L: 61 3L-: 20	1, 2, 3
Ma et al. (13)	Multi-center RCT	II	65/63	Pyr-Cap	Lap-Cap	Adju 37 meta 38	1L: 37 2L-: 38	1
Jiang et al. (37)	Multi-center RCT	III	185/94	Pyr-Cap	Cap	Adju 125 meta 177	1L: 125 2L-: 177	1, 3
Emens et al. (39)	Multi-center RCT	II	133/69	Ate-T-DM1	T-DM1	NA	NA	1, 2, 3, 4
Xu et al. (38)	Multi-center RCT	III	134/132	Pyr-Cap	Lap-Cap	Adju 168 meta 138	1L: 168 2L-: 138	1, 2, 3, 4
Saura et al. (12)	Multi-center RCT	III	307/314	Ner-Cap	Lap-Cap	meta 621	3L-: 621	1, 2, 3

†Main outcomes: 1, progression-free survival; 2, overall survival; 3, overall response rate; 4, grade ≥3 adverse events.

RCT, randomized clinical trial; Lap, lapatinib; Cap, capecitabine; Tra, trastuzumab; T-DM1, trastuzumab emtansine; Ner, neratinib; Per, pertuzumab; Pyr, pyrotinib; Ate, atezolizumab; adju, adjuvant; meta, metastatic; 1L, first line; 2L, second line; 3L, third line, NA, not available.

### Risk of Bias Assessment

**Figure 2:**
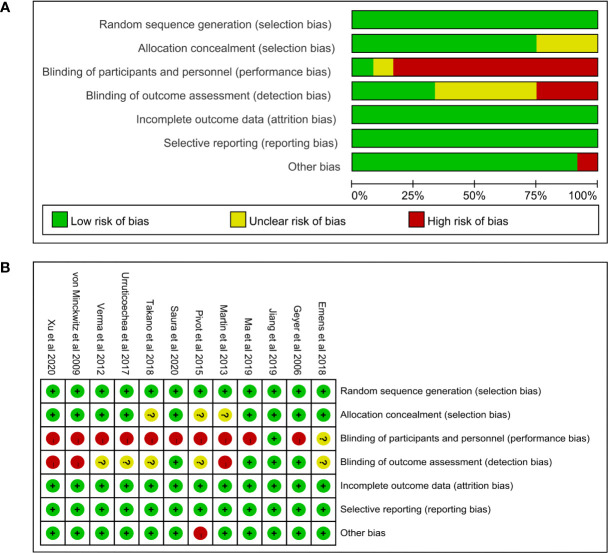
**(A)** Risk of bias graph and **(B)** summary. In the risk of bias graph, the length of each rectangle was proportional to the number of studies being assessed as corresponding risk of bias. In the risk of bias summary, the risk of bias of each domain in each included study was listed.

The risk of bias graph and the risk of bias summary were presented in **Figure 2**. Only one study was judged to be at low risk of bias (37), one study was judged to be at unclear risk of bias (39), and ten studies were judged to be at high risk of bias (8–13, 34–36, 38). All studies used random sequence generation to generate allocation sequences. Nine studies used appropriate allocation and concealment methods (8–10, 12, 13, 34, 37–39), but the other three studies did not state details (11, 35, 36). The major concern is that the ten studies with high risk of bias are open-label trials and no blinding of participants and personnel was performed (8–13, 34–36, 38). In addition, one study was judged to be at high risk in the domain of other bias because of early study termination (11).

### Network Meta-Analysis

**Figure 3:**
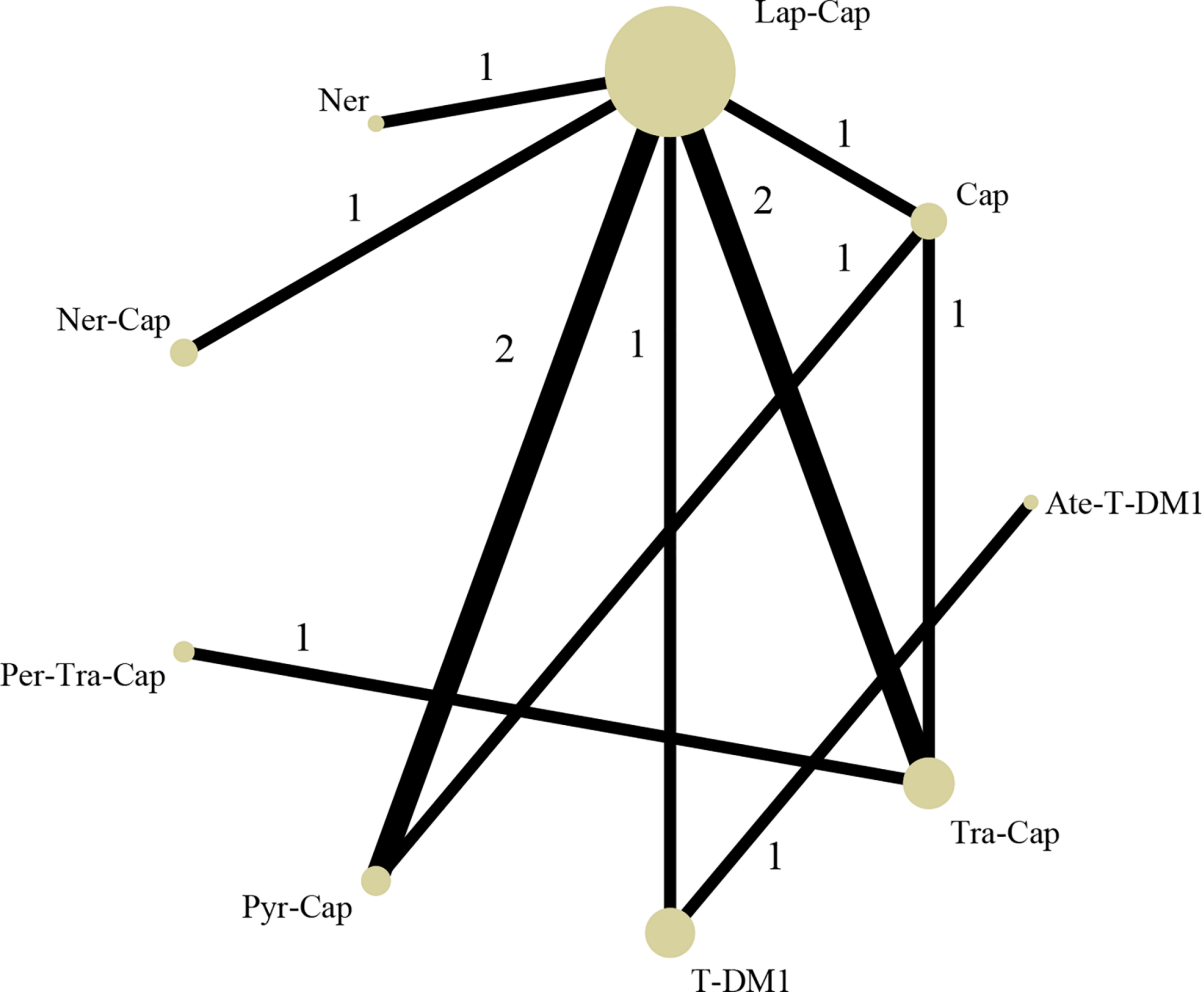
Network structure diagram. T-DM1, trastuzumab emtansine; Lap, lapatinib; Tra, trastuzumab; Cap, capecitabine; Ner, neratinib; Per, pertuzumab; Pyr, pyrotinib; Ate, atezolizumab.

The network map among nine treatment strategies (T-DM1, Lap-Cap, Tra-Cap, Cap, Ner, Per-Tra-Cap, Pyr-Cap, atezolizumab (Ate; Tecentriq™, Roche)-T-DM1, and Ner-Cap) was plotted in Stata 15.0 (**Figure 3**) using Network package. Lap-Cap and Tra-Cap ranked in the top two among all regimens in the number of trials and the number of randomized participants. The original data (PFS, OS, ORR, and grade ≥3 AEs) were presented in **Appendix 2**.

### Progression-Free Survival

**Figure 4:**
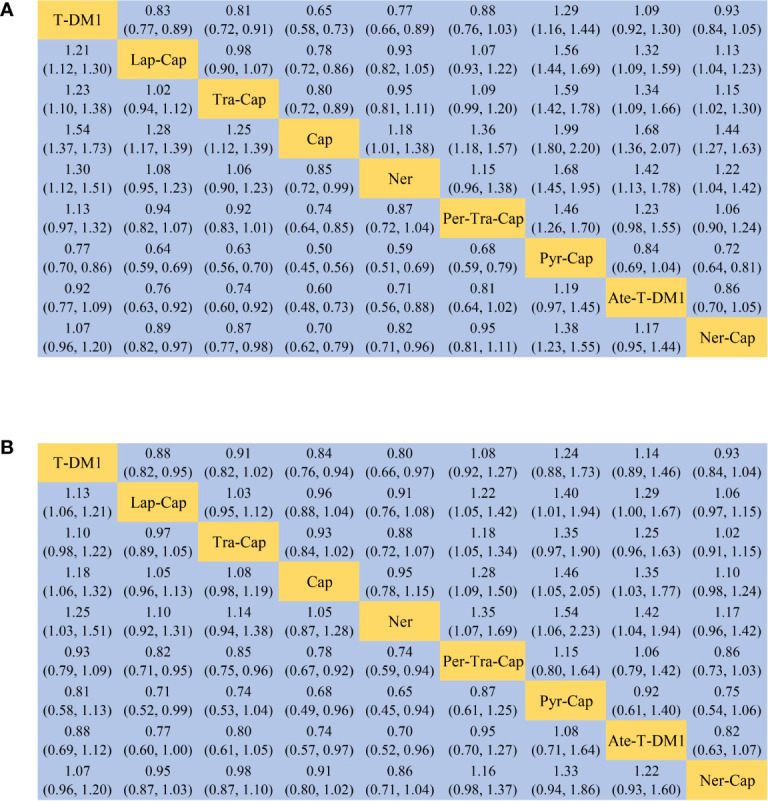
League table of network meta-analysis of the nine anti-HER2 regimens. **(A)** PFS and **(B)** OS. PFS, progression-free survival; OS, overall survival; T-DM1, trastuzumab emtansine; Lap, lapatinib; Tra, trastuzumab; Cap, capecitabine; Ner, neratinib; Per, pertuzumab; Pyr, pyrotinib; Ate, atezolizumab.

**Figure 5:**
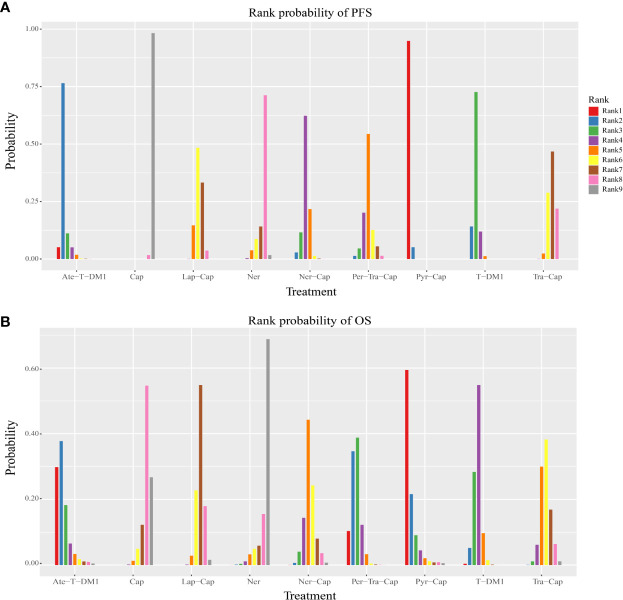
Rank probability plot of **(A)** PFS and **(B)** OS. PFS, progression-free survival; OS, overall survival; T-DM1, trastuzumab emtansine; Lap, lapatinib; Tra, trastuzumab; Cap, capecitabine; Ner, neratinib; Per, pertuzumab; Pyr, pyrotinib; Ate, atezolizumab.

**Figure 6:**
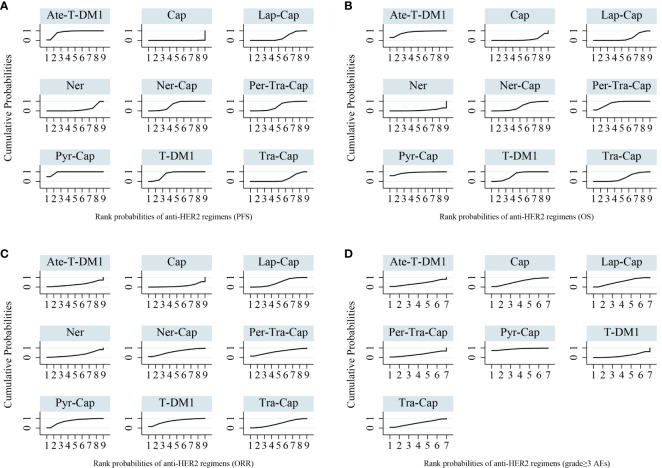
Surface under the cumulative ranking curves (SUCRA) for anti-HER2 regimens of all outcomes. **(A)** PFS **(B)** OS **(C)** ORR **(D)** grade ≥3 AEs. PFS, progression-free survival; OS, overall survival; ORR, overall response rate; AEs, adverse events; T-DM1, trastuzumab emtansine; Lap, lapatinib; Tra, trastuzumab; Cap, capecitabine; Ner, neratinib; Per, pertuzumab; Pyr, pyrotinib; Ate, atezolizumab.

All of the twelve studies reported PFS data (8–13, 34–39). As shown in **Figure 4A**, Pyr-Cap was significantly superior in PFS than T-DM1 (HR, 95%CI: 0.77, 0.70–0.86), Lap-Cap (0.64, 0.59–0.69), Tra-Cap (0.63, 0.56–0.70), Cap (0.50, 0.45–0.56), Ner (0.59, 0.51–0.69), Per-Tra-Cap (0.68, 0.59–0.79), and Ner-Cap (0.72, 0.64–0.81). T-DM1 was significantly superior than Lap-Cap (HR, 95%CI: 0.83, 0.77–0.89), Tra-Cap (0.81, 0.72–0.91), Cap (0.65, 0.58–0.73), and Ner (0.77, 0.66–0.89). Ate-T-DM1 was significantly superior than Lap-Cap (HR, 95%CI: 0.76, 0.63–0.92), Tra-Cap (0.74, 0.60–0.92), Cap (0.60, 0.48–0.73), and Ner (0.71, 0.56–0.88). Ner-Cap was significantly superior than Lap-Cap (HR, 95%CI: 0.89, 0.82–0.97), Tra-Cap (0.87, 0.77–0.98), Cap (0.70, 0.62–0.79), and Ner (0.82, 0.71–0.96). All regimens were significantly superior in PFS than Cap (T-DM1 (HR, 95%CI: 0.65, 0.58–0.73), Lap-Cap (0.78, 0.72–0.86), Tra-Cap (0.80, 0.72–0.89), Ner (0.85, 0.72–0.99), Per-Tra-Cap (0.74, 0.64–0.85), Pyr-Cap (0.50, 0.45–0.56), Ate-T-DM1 (0.60, 0.48–0.73), and Ner-Cap (0.70, 0.62–0.79)). The rank probability plot of PFS was presented in **Figure 5A**. Pyr-Cap had the highest probability to rank as the best regimen, while Cap had the highest probability to be the worst one. In addition, we calculated the values of SUCRA to obtain a more accurate ranking (**Figure 6A**). According to the outcomes of SUCRA (99.4%) and MeanRank (1.1), Pyr-Cap indeed had the highest probability to be the best treatment, followed by Ate-T-DM1 (84.6%, 2.2), T-DM1 (75.0%, 3.0), Ner-Cap (61.5%, 4.1), Per-Tra-Cap (50.7%, 4.9), Lap-Cap (34.3%, 6.3), Tra-Cap (26.5%, 6.9), Ner (17.9%, 7.6), and Cap (0.2%, 9.0) (**Appendix 3.1**).

### Overall Survival

For OS, ten studies reported relevant data (8–12, 34–36, 38, 39). As shown in **Figure 4B**, Pyr-Cap was significantly superior in OS than Lap-Cap (HR, 95%CI: 0.71, 0.52–0.99), Cap (0.68, 0.49–0.96), and Ner (0.65, 0.45–0.94). T-DM1 was significantly superior than Lap-Cap (HR, 95%CI: 0.88, 0.82–0.95), Cap (0.84, 0.76–0.94), and Ner (0.80, 0.66–0.97). Per-Tra-Cap was significantly superior than Lap-Cap (HR, 95%CI: 0.82, 0.71–0.95), Tra-Cap (0.85, 0.75–0.96), Cap (0.78, 0.67–0.92), and Ner (0.74, 0.59–0.94). Ate-T-DM1 was significantly superior than Cap (HR, 95%CI: 0.74, 0.57–0.97) and Ner (0.70, 0.52–0.96). According to the rank probability plot of OS (**Figure 5B**), Pyr-Cap had the highest probability to rank as the best regimen, while Ner had the highest probability to rank as the worst one. The cumulative probabilities of all regimens were shown in **Figure 6B** and the ranking results of SUCRA are as follows: Pyr-Cap (89.7%, 1.8), Ate-T-DM1 (83.4%, 2.3), Per-Tra-Cap (79.3%, 2.7), T-DM1 (65.9%, 3.7), Ner-Cap (46.3%, 5.6), Tra-Cap (39.1%, 5.9), Lap-Cap (26.0%, 6.9), Cap (12.5%, 8.0), and Ner (8.0%, 8.4) (**Appendix 3.2**).

The DIC values of fixed effects model were lower than that of random effects model in PFS (−24.69 *vs.* −23.11), as well as in OS (−13.88 *vs.* −13.47). In order to minimize the magnitude of inconsistency and ensure the stability of the results, we chose the fixed effects model to analyze PFS and OS data in this network meta-analysis. The results of PFS and OS by random effects model were shown in **Appendix 4**. 

### Overall Response Rate

Ten studies evaluated ORR (8–10, 12, 34–39). Pyr was significantly superior than Cap (OR, 95%CI: 7.87, 1.22–56.51) in ORR (**Appendix 5A**). The rank probability plot of ORR indicated that Pyr-Cap had the highest probability to be the best regimen and Cap was most likely the worst one (**Appendix 6A**). The cumulative probabilities of all regimens were shown in **Figure 6C** and the ranking results of SUCRA are as follows: Pyr-Cap (86.4%, 2.1), T-DM1 (72.5%, 3.3), Per-Tra-Cap (60.6%, 4.2), Ner-Cap (60.6%, 4.2), Lap-Cap (48.8%, 5.1), Tra-Cap (47.7%, 5.2), Ner (30.6%, 6.5), Ate-T-DM1 (29.0%, 6.7), and Cap (14.0%, 7.9) (**Appendix 3.3**). 

### Grade ≥3 Adverse Events

Only six studies evaluated grade ≥3 AEs (8–10, 34, 38, 39). No significant difference was found in any comparison in grade ≥3 AEs (**Appendix 5B**). Pyr-Cap had the highest probability to be the regimen with most frequent grade ≥3 AEs, while T-DM1 most likely had the lowest incidence of grade ≥3 AEs according to the rank probability plot (**Appendix 6B**). The cumulative probabilities of seven regimens were shown in **Figure 6D** and the ranking results of SUCRA are as follows: Pyr-Cap (89.3%, 1.6), Cap (56.3%, 3.5), Lap-Cap (55.2%, 3.7), Tra-Cap (51.7%, 3.9), Ate-T-DM1 (39.7%, 4.6), Per-Tra-Cap (34.0%, 4.9), and T-DM1 (21.2%, 5.7) (**Appendix 3.4**).

Both the inconsistency factors in the results of ORR and grade ≥3 AEs were close to 0. In addition, the random effects standard deviations were roughly equal between the consistency model and inconsistency model (median 0.67, 95%CI [0.08–2.18] *vs.* 0.64, [0.01–2.23] for ORR; 0.47, [0.03–0.93] *vs.*. 0.49, [0.04–0.93] for grade ≥3 AEs). Therefore, we could confirm that the data are consistent. 

## Discussion

MBC accounts for 6% of all BC, and approximately 30% early BC eventually develop metastatic disease (40). For patients with MBC, which is incurable with currently available therapies, the main therapeutic goals are to prolong PFS and reduce the incidence of AEs (41). Combining Tra with first-line chemotherapy (taxane in most cases) has been shown to improve PFS and OS among patients with HER2+ MBC (42, 43). For patients who progressed on Tra and a taxane, T-DM1 was recommended in the USA based on the results of EMILIA, while Pyr had better applicability in China (9, 13). Due to the lack of head-to-head comparisons of efficacy and safety between T-DM1 and Pyr, network meta-analysis combining direct and indirect evidence seems necessary and may be instructive for further studies.

A previous network meta-analysis by Paracha et al. compared the efficacy and safety of various anti-HER2 regimens in HER2+ MBC patients who progressed on prior taxane/Tra (44). Seven RCTs with 2857 subjects were included. Six regimens including T-DM1, Lap-Cap, Tra-Cap, Cap, Ner, and Per-Tra-Cap were included in the model. The results indicated that T-DM1 were generally favorable compared with other regimens both in efficacy and tolerability profiles (44). In present network meta-analysis, we added additional data from five updated studies (12, 13, 37–39) and compared the efficacy and safety of nine anti-HER2 regimens (T-DM1, Lap-Cap, Tra-Cap, Cap, Ner, Per-Tra-Cap, Pyr-Cap, Ate-T-DM1, and Ner-Cap), focusing on the comparison between T-DM1 and Pyr-Cap. A total of twelve RCTs containing 4353 HER2+ MBC patients pre-treated with Tra and a taxane were included. The results indicated that Pyr-Cap was more favorable in PFS than T-DM1, Lap-Cap, Tra-Cap, Cap, Ner, Per-Tra-Cap, and Ner-Cap. For OS, Pyr-Cap showed further improvement than Lap-Cap, Cap, and Ner. For ORR, Pyr-Cap was significantly superior than Cap. No significant difference was observed in grade ≥3 AEs. The SUCRA is a numerical representation of the ranking probability of each intervention in efficacy or safety, providing researchers with preliminary judgments on the ranking of interventions. With SUCRA, researchers could simplify the process of evaluating the efficacy of interventions by converting large amounts of data into simple ranking probabilities (25). In this study, Pyr-Cap ranked in the highest in PFS, OS, ORR, and grade ≥3 AEs according to the values of SUCRA.

As a novel irreversible tyrosine kinase inhibitor, Pyr has shown clinically significant benefits and acceptable tolerance in phase I/II studies (13, 21). Pyr exerts its anti-HER2 effects *via* a completely different mechanism from Tra. It directly acts on the intracellular tyrosine kinase region and blocks the downstream pathways of HER family homo/heterodimers (20). Therefore, Pyr may be still effective for HER2+ MBC patients who progressed on Tra (45). The PHENIX study by Jiang et al. is a randomized, double-blinded, controlled trial (37). 185 and 94 HER2+ MBC patients pre-treated with Tra and a taxane were randomly assigned to receive Pyr-Cap and Cap, respectively. Compared with Cap, Pyr-Cap significantly prolonged the PFS (11.1 vs. 4.1 months; HR, 95%CI: 0.18, 0.13–0.26). The PHOEBE study by Xu et al. reported in ASCO 2020 is an open-label randomized phase III trial (38, 46). 134 and 132 HER2+ MBC patients pre-treated with Tra and a taxane were randomly assigned to receive Pyr-Cap and Lap-Cap, respectively. Patients treated with Pyr-Cap showed significantly improved PFS compared with those treated with Lap-Cap (12.5 vs. 6.8 months; HR, 95%CI: 0.39, 0.27–0.56). However, the PFS of patients who received T-DM1 in the EMILIA study was only 9.6 months (9). These results confirmed the therapeutic effects of Pyr in later treatment lines of HER2+ MBC patients, underlining the importance of using anti-HER2 agents with different mechanisms after progressing on Tra. In addition to the different anti-tumor mechanisms, a major difference between Pyr and T-DM1 is the common grade ≥3 AEs. In EMILIA study, thrombocytopenia (12.9%), elevated serum concentrations of aspartate aminotransferase (4.3%), and alanine aminotransferase (2.9%) were the most commonly reported grade ≥3 AEs of T-DM1 (9). Nevertheless, the most frequent grade ≥3 AEs of Pyr-Cap were diarrhea (30.8%) and hand-foot syndrome (15.7%) in the PHENIX study, and diarrhea (30.6%) and hand-foot syndrome (16.4%) in the PHOEBE study (37, 38, 46). Frequent occurrence of diarrhea could be the reason why Pyr-Cap ranked in the highest of grade ≥3 AEs in this network meta-analysis. Despite the high incidence, the grade ≥3 diarrhea caused by Pyr-Cap is reversible and occurs in the early stage of treatment with a short duration, barely leading to the discontinuation of treatment (37, 38, 46). Therefore, the safety of Pyr could be considered acceptable. From a cost-effective point of view, Pyr is currently more cost-effective than T-DM1. In China, one therapy cycle of Pyr costs about CNY 9,030/USD 1,398, which is much less than T-DM1 (CNY 36,000/USD 5,572). Also, as the ease of use of Pyr, patients who go to other places for medical treatment do not need to travel frequently between hospital and home, further reducing the economic and time costs.

To our knowledge, this is the first network meta-analysis containing the comparisons of the efficacy and safety between Pyr and T-DM1 in HER2+ MBC patients pre-treated with Tra and a taxane. Rigorous methodology was used and a large number of studies and patients were included, increasing the reliability of this current study. However, there are a few limitations that should be mentioned. First, nine of twelve studies were judged to be at high risk, which may affect the validity of the results. Second, despite the advantage of enabling indirect comparisons, the evidence level of network meta-analysis is inferior to traditional pare-wise meta-analysis. At last, no study has directly compared the efficacy and safety between Pyr and T-DM1. Therefore, the results of this network meta-analysis need to be verified in further high-quality RCTs. 

## Conclusions

In conclusion, Pyr may be more effective than T-DM1 in HER2+ MBC patients pre-treated with Tra and a taxane. However, it may be associated with more grade≥3 AEs.

## Data Availability Statement

The original contributions presented in the study are included in the article/**Supplementary Material**. Further inquiries can be directed to the corresponding author.

## Author Contributions

HaL and WH searched the literatures, conducted the statistical analysis, and wrote the manuscript. YL and WP extracted the data and assessed the risk of bias. HuL conducted the statistical analysis and revised the manuscript. All authors contributed to the article and approved the submitted version.

## Conflict of Interest

The authors declare that the research was conducted in the absence of any commercial or financial relationships that could be construed as a potential conflict of interest.

## Supplementary Material

Appendix 5 League table of network meta-analysis of the nine anti-HER2 regimens. **(A)** ORR and **(B)** grade ≥3 adverse events. ORR, overall response rate; T-DM1, trastuzumab emtansine; Lap, lapatinib; Tra, trastuzumab; Cap, capecitabine; Ner, neratinib; Per, pertuzumab; Pyr, pyrotinib; Ate, atezolizumab.

Appendix 6 Rank probability plot of **(A)** ORR and **(B)** grade ≥3 adverse events. A, T-DM1; B, Lap- Cap; C, Tra-Cap; D, Cap; E, Ner; F, Per-Tra-Cap; G, Pyr-Cap; H, Ate-T-DM1; I, Ner-Cap; ORR, overall response rate; T-DM1, trastuzumab emtansine; Lap, lapatinib; Tra, trastuzumab; Cap, capecitabine; Ner, neratinib; Per, pertuzumab; Pyr, pyrotinib; Ate, atezolizumab.
