# Supplementary material for: Efficacy and Safety of Pyrotinib Versus T-DM1 in HER2+ Metastatic Breast Cancer Patients Pre-Treated With Trastuzumab and a Taxane: A Bayesian Network Meta-Analysis
Source: Front Oncol. 2021 May 3;11:608781. doi: 10.3389/fonc.2021.608781 (PMC8127838; doi:10.3389/fonc.2021.608781)
Supplement: Supplementary file 2 [file Table_1.docx]

**Main search algorithm:**

(breast OR mammary) AND (cancer OR neoplasm OR oncology OR tumor OR malignancy OR carcinoma OR adenocarcinoma OR sarcoma) AND (metastasis OR metastatic OR advanced OR secondary OR recurrent OR inoperable OR unresectable OR disseminated OR incurable) AND (“human epidermal growth factor receptor 2” OR HER2 OR HER-2 OR Her-2 OR ERBB2 OR neu) AND (positive OR enriched OR overexpressing OR overexpressed) AND (trial OR study) AND (randomized OR randomized OR randomly OR randomization OR RCT) AND (trastuzumab OR Herceptin) AND (Anti-HER2 OR HER2-targeted OR lapatinib OR tykerb OR neratinib OR pyrotinib OR pertuzumab OR T-DM1 OR “trastuzumab emtansine” OR trastuzumab-DM1 OR trastuzumab-MCC-DM1 OR margetuximab OR tucatinib OR “trastuzumab deruxtecan” OR DS8201 OR poziotinib OR afatinib OR everolimus OR chemotherapy OR cyclophosphamide OR methotrexate OR fluorouracil OR 5FU OR 5-FU OR doxorubicin OR mitoxantrone OR epirubicin OR paclitaxel OR docetaxel OR liposomal doxorubicin OR nab-paclitaxel OR “nab paclitaxel” OR eribulin OR capecitabine OR vinorelbine OR carboplatin OR cisplatin OR platinum OR gemcitabine)

**Sources:**

**2767 Total**

559 Pubmed

901 Web of Science

1264 Embase

3 ASCO+ESMO

**1636 after duplications removed** (1131 duplications were removed)

**67 after screening of titles and abstracts (**1569 records were excluded for the following reasons: irrelevant studies, reviews, comments, case-reports, letters, conference abstracts, non-human studies, and other types of tumors)

**12 after further evaluation** (55 records excluded for the following reasons: non-RCT studies, n=21; non-English studies, n=15; patients only received first-line treatment, n=16; patients pretreated with ≥ 3-line anti-HER2 treatment, n=3)

**12 included in network meta-analysis after full-text assessment for eligibility**
