# Supplementary material for: Efficacy and Safety of Pyrotinib Versus T-DM1 in HER2+ Metastatic Breast Cancer Patients Pre-Treated With Trastuzumab and a Taxane: A Bayesian Network Meta-Analysis
Source: Front Oncol. 2021 May 3;11:608781. doi: 10.3389/fonc.2021.608781 (PMC8127838; doi:10.3389/fonc.2021.608781)
Supplement: Supplementary file 3 [file Table_2.docx]

| **Appendix 2** Original data of included studies | | | | | | | |
| --- | --- | --- | --- | --- | --- | --- | --- |
| **Author** | **Year** | **Treatment** | **Control** | **PFS (months)**  **HR (95%CI)** | **OS (months)**  **HR (95%CI)** | **ORR** | **Grade≥ 3 AEs** |
| Geyer et al | 2006 | Lap-Cap | Cap | 6.2 vs. 4.3 0.57 (0.43-0.77) | 75.0 vs. 64.7 0.87 (0.71-1.08) | 47/198 vs. 28/201 23.7% vs 13.9% | T: 12/198 C: 12/191 |
| von Minckwitz et al | 2009 | Tra-Cap | Cap | 8.2 vs. 5.6 0.69 (0.48-0.97) | 24.9 vs 20.6 0.94 (0.65-1.35) | 37/77 vs. 20/74 48.1% vs 27.0% | T: 49/77 C: 49/74 |
| Verma et al | 2012 | T-DM1 | Lap-Cap | 9.6 vs. 6.4 0.65 (0.55-0.77) | 29.9 vs 25.9 0.75 (0.64-0.88) | 173/397 vs. 120/389 43.6% vs 30.8% | T: 233/490 C: 291/488 |
| Martin et al | 2013 | Ner | Lap-Cap | 4.5 vs. 6.8  1.19 (0.89-1.60) | 19.7 vs. 23.6 1.25 (0.83-1.86) | 34/117 vs. 47/116 29.1% vs 40.5% | NA |
| Pivot et al | 2015 | Lap-Cap | Tra-Cap | 6.6 vs. 6.1 1.13 (0.85-1.50) | 22.7 vs. 27.3 1.18 (0.76-1.18) | NA | NA |
| Urruticoechea et al | 2017 | Per-Tra-Cap | Tra-Cap | 28.6 vs. 25.3 0.82 (0.65-1.02) | 28.1 vs. 36.1 0.68 (0.51-0.90) | 73/163 vs. 61/164 44.8% vs 37.2% | T: 118/228 C: 130/218 |
| Takano et al | 2018 | Lap-Cap | Tra-Cap | 7.1 vs. 6.1 0.81 (0.55-1.21) | NA vs. 31.0 0.58 (0.26-1.31) | 16/40 vs. 15/37 40.0% vs 40.5% | NA |
| Ma et al | 2019 | Pyr-Cap | Lap-Cap | 18.1 vs. 7.0 0.37 (0.19-0.74) | NA | NA | NA |
| Jiang et al | 2019 | Pyr-Cap | Cap | 11.1 vs. 4.1 0.18 (0.13-0.26) | NA | 127/185 vs. 15/94 68.6% vs 16.0% | NA |
| Emens et al | 2019 | Ate-T-DM1 | T-DM1 | 8.2 vs. 6.8 0.82 (0.55-1.23) | NA vs. NA 0.74 (0.42-1.30) | 45/133 vs. 43/69 33.8% vs 62.3% | T: 70/133 C: 31/69 |
| Xu et al | 2020 | Pyr-Cap | Lap-Cap | 12.5 vs. 6.8 0.39 (0.27-0.56) | NA vs. NA  0.46 (0.22-0.99) | 90/134 vs. 68/132 67.2% vs 51.5% | T: 77/134 C: 45/132 |
| Saura et al | 2020 | Ner-Cap | Lap-Cap | 8.8 vs. 6.6  0.76 (0.63-0.93) | 24.0 vs. 22.2 0.88 (0.72-1.07) | 84/256 vs. 72/270 32.8% vs 26.7% | NA |

**Abbreviations:** T-DM1, trastuzumab emtansine; Lap, lapatinib; Tra, trastuzumab; Cap, capecitabine; Ner, neratinib; Per, pertuzumab; Pyr, pyrotinib; Ate, atezolizumab; PFS, progression-free survival; OS, overall survival; HR, hazard ratio; CI, confidence interval; ORR, overall response rate; AEs, adverse events; T, treatment; C, control; NA, not available
