# Supplementary material for: Efficacy and Safety of Pyrotinib Versus T-DM1 in HER2+ Metastatic Breast Cancer Patients Pre-Treated With Trastuzumab and a Taxane: A Bayesian Network Meta-Analysis
Source: Front Oncol. 2021 May 3;11:608781. doi: 10.3389/fonc.2021.608781 (PMC8127838; doi:10.3389/fonc.2021.608781)
Supplement: Supplementary file 4 [file Table_3.docx]

| Treatment | SUCRA | PrBest | MeanRank |
| --- | --- | --- | --- |
| T-DM1 | 75.0 | 0.0 | 3.0 |
| Lap-Cap | 34.3 | 0.0 | 6.3 |
| Tra-Cap | 26.5 | 0.0 | 6.9 |
| Cap | 0.2 | 0.0 | 9.0 |
| Ner | 17.9 | 0.0 | 7.6 |
| Per-Tra-Cap | 50.7 | 0.0 | 4.9 |
| Pyr-Cap | 99.4 | 94.9 | 1.1 |
| Ate-T-DM1 | 84.6 | 5.1 | 2.2 |
| Ner-Cap | 61.5 | 0.0 | 4.1 |

**Appendix 3.1** SUCRA summary of different anti-HER2 regimens (PFS)

**Appendix 3.2** SUCRA summary of different anti-HER2 regimens (OS)

| Treatment | SUCRA | PrBest | MeanRank |
| --- | --- | --- | --- |
| T-DM1 | 65.9 | 0.4 | 3.7 |
| Lap-Cap | 26.0 | 0.0 | 6.9 |
| Tra-Cap | 39.1 | 0.0 | 5.9 |
| Cap | 12.5 | 0.0 | 8.0 |
| Ner | 8.0 | 0.0 | 8.4 |
| Per-Tra-Cap | 79.3 | 10.4 | 2.7 |
| Pyr-Cap | 89.7 | 59.4 | 1.8 |
| Ate-T-DM1 | 83.4 | 29.8 | 2.3 |
| Ner-Cap | 46.3 | 0.0 | 5.6 |

**Appendix 3.3** SUCRA summary of different anti-HER2 regimens (ORR)

| Treatment | SUCRA | PrBest | MeanRank |
| --- | --- | --- | --- |
| T-DM1 | 72.5 | 16.0 | 3.3 |
| Lap-Cap | 48.8 | 0.0 | 5.1 |
| Tra-Cap | 47.7 | 2.0 | 5.2 |
| Cap | 14.0 | 0.0 | 7.9 |
| Ner | 30.6 | 3.0 | 6.5 |
| Per-Tra-Cap | 60.6 | 15.0 | 4.2 |
| Pyr-Cap | 86.4 | 51.0 | 2.1 |
| Ate-T-DM1 | 29.0 | 3.0 | 6.7 |
| Ner-Cap | 60.6 | 10.0 | 4.2 |

**Appendix 3.4** SUCRA summary of different anti-HER2 regimens (grade≥3 AEs)

| Treatment | SUCRA | PrBest | MeanRank |
| --- | --- | --- | --- |
| T-DM1 | 21.2 | 0 | 5.7 |
| Lap-Cap | 55.2 | 1 | 3.7 |
| Tra-Cap | 51.7 | 8 | 3.9 |
| Cap | 56.3 | 5 | 3.5 |
| Per-Tra-Cap | 34 | 6 | 4.9 |
| Pyr-Cap | 89.3 | 73 | 1.6 |
| Ate-T-DM1 | 39.7 | 6 | 4.6 |

**Abbreviations:** SUCRA, surface under cumula­tive ranking probability curve; HER2, human epidermal growth factor receptor 2-positive; T-DM1, trastuzumab emtansine; Lap, lapatinib; Tra, trastuzumab; Cap, capecitabine; Ner, neratinib; Per, pertuzumab; Pyr, pyrotinib; Ate, atezolizumab; PFS, progression-free survival; OS, overall survival; ORR, overall response rate; AEs, adverse events
