# Supplementary material for: Efficacy and Safety of Pyrotinib Versus T-DM1 in HER2+ Metastatic Breast Cancer Patients Pre-Treated With Trastuzumab and a Taxane: A Bayesian Network Meta-Analysis
Source: Front Oncol. 2021 May 3;11:608781. doi: 10.3389/fonc.2021.608781 (PMC8127838; doi:10.3389/fonc.2021.608781)
Supplement: Appendix 5 — League table of network meta-analysis of the nine anti-HER2 regimens. (A) ORR and (B) grade ≥3 adverse events. ORR, overall response rate; T-DM1, trastuzumab emtansine; Lap, lapatinib; Tra, trastuzumab; Cap, capecitabine; Ner, neratinib; Per, pertuzumab; Pyr, pyrotinib; Ate, atezolizumab. [file Image_1.pdf]

(A)

T-DM1	1.71 (0.15, 20.17)	1.80 (0.08, 41.04)	4.69 (0.26, 93.58)	2.90 (0.10, 96.04)	1.30 (0.03, 62.42)	0.61 (0.03, 11.51)	3.21 (0.29, 36.86)	1.29 (0.04, 40.47)
0.58 (0.05, 6.54)	Lap-Cap	1.03 (0.16, 7.20)	2.75 (0.48, 15.88)	1.64 (0.15, 19.25)	0.73 (0.04, 17.44)	0.36 (0.05, 2.38)	1.86 (0.06, 59.10)	0.75 (0.06, 8.01)
0.56 (0.02, 12.27)	0.97 (0.14, 6.14)	Tra-Cap	2.69 (0.36, 18.02)	1.60 (0.07, 33.45)	0.72 (0.07, 7.99)	0.35 (0.03, 3.60)	1.78 (0.03, 86.87)	0.73 (0.03, 13.05)
0.21 (0.01, 3.84)	0.36 (0.06, 2.06)	0.37 (0.06, 2.77)	Cap	0.59 (0.03, 11.92)	0.27 (0.01, 6.47)	0.13 (0.02, 0.82)	0.68 (0.02, 29.86)	0.27 (0.01, 5.03)
0.34 (0.01, 10.20)	0.61 (0.05, 6.89)	0.63 (0.03, 15.37)	1.69 (0.08, 32.75)	Ner	0.44 (0.01, 25.41)	0.21 (0.01, 4.83)	1.13 (0.01, 73.16)	0.45 (0.02, 12.92)
0.77 (0.02, 35.59)	1.36 (0.06, 25.88)	1.38 (0.13, 13.49)	3.71 (0.15, 74.98)	2.25 (0.04, 94.45)	Per-Tra-Cap	0.48 (0.01, 12.90)	2.44 (0.03, 225.71)	1.02 (0.02, 37.77)
1.64 (0.09, 36.51)	2.81 (0.42, 20.43)	2.89 (0.28, 39.75)	7.87 (1.22, 56.51)	4.67 (0.21, 104.34)	2.10 (0.08, 70.65)	Pyr-Cap	5.35 (0.10, 271.31)	2.13 (0.09, 52.51)
0.31 (0.03, 3.47)	0.54 (0.02, 16.14)	0.56 (0.01, 29.46)	1.47 (0.03, 65.24)	0.88 (0.01, 67.58)	0.41 (0.01, 39.63)	0.19 (0.01, 9.73)	Ate-T-DM1	0.41 (0.01, 26.02)
0.78 (0.02, 24.22)	1.33 (0.12, 15.83)	1.36 (0.08, 30.67)	3.70 (0.20, 82.82)	2.21 (0.08, 62.85)	0.98 (0.03, 54.72)	0.47 (0.02, 11.39)	2.46 (0.04, 200.77)	Ner-Cap

(B)

T-DM1	0.52 (0.16, 1.85)	0.55 (0.05, 5.61)	0.50 (0.08, 3.18)	0.76 (0.05, 11.10)	0.20 (0.03, 1.24)	0.74 (0.18, 2.67)
1.93 (0.54, 6.37)	Lap-Cap	1.05 (0.14, 7.33)	0.95 (0.23, 3.73)	1.46 (0.13, 15.99)	0.38 (0.11, 1.40)	1.42 (0.24, 8.34)
1.82 (0.18, 20.95)	0.95 (0.14, 7.11)	Tra-Cap	0.89 (0.23, 3.60)	1.37 (0.38, 4.90)	0.37 (0.04, 4.10)	1.35 (0.09, 21.48)
2.01 (0.31, 13.25)	1.05 (0.27, 4.44)	1.12 (0.28, 4.37)	Cap	1.52 (0.22, 9.46)	0.41 (0.06, 2.86)	1.50 (0.15, 14.89)
1.32 (0.09, 20.89)	0.68 (0.06, 7.49)	0.73 (0.20, 2.61)	0.66 (0.11, 4.45)	Per-Tra-Cap	0.27 (0.02, 4.05)	0.99 (0.05, 21.85)
4.97 (0.81, 29.74)	2.60 (0.71, 9.33)	2.71 (0.24, 26.91)	2.45 (0.35, 16.36)	3.76 (0.25, 54.25)	Pyr-Cap	3.70 (0.37, 32.69)
1.34 (0.38, 5.42)	0.70 (0.12, 4.08)	0.74 (0.05, 10.92)	0.67 (0.07, 6.76)	1.01 (0.05, 21.17)	0.27 (0.03, 2.72)	Ate-T-DM1

T-DM1, trastuzumab emtansine; Lap, lapatinib; Tra, trastuzumab; Cap, capecitabine; Ner, neratinib; Per, pertuzumab; Pyr, pyrotinib; Ate, atezolizumab
