# Supplementary material for: Efficacy and Safety of Pyrotinib Versus T-DM1 in HER2+ Metastatic Breast Cancer Patients Pre-Treated With Trastuzumab and a Taxane: A Bayesian Network Meta-Analysis
Source: Front Oncol. 2021 May 3;11:608781. doi: 10.3389/fonc.2021.608781 (PMC8127838; doi:10.3389/fonc.2021.608781)
Supplement: Appendix 6 — Rank probability plot of (A) ORR and (B) grade ≥3 adverse events. A, T-DM1; B, Lap- Cap; C, Tra-Cap; D, Cap; E, Ner; F, Per-Tra-Cap; G, Pyr-Cap; H, Ate-T-DM1; I, Ner-Cap; ORR, overall response rate; T-DM1, trastuzumab emtansine; Lap, lapatinib; Tra, trastuzumab; Cap, capecitabine; Ner, neratinib; Per, pertuzumab; Pyr, pyrotinib; Ate, atezolizumab. [file Image_2.pdf]

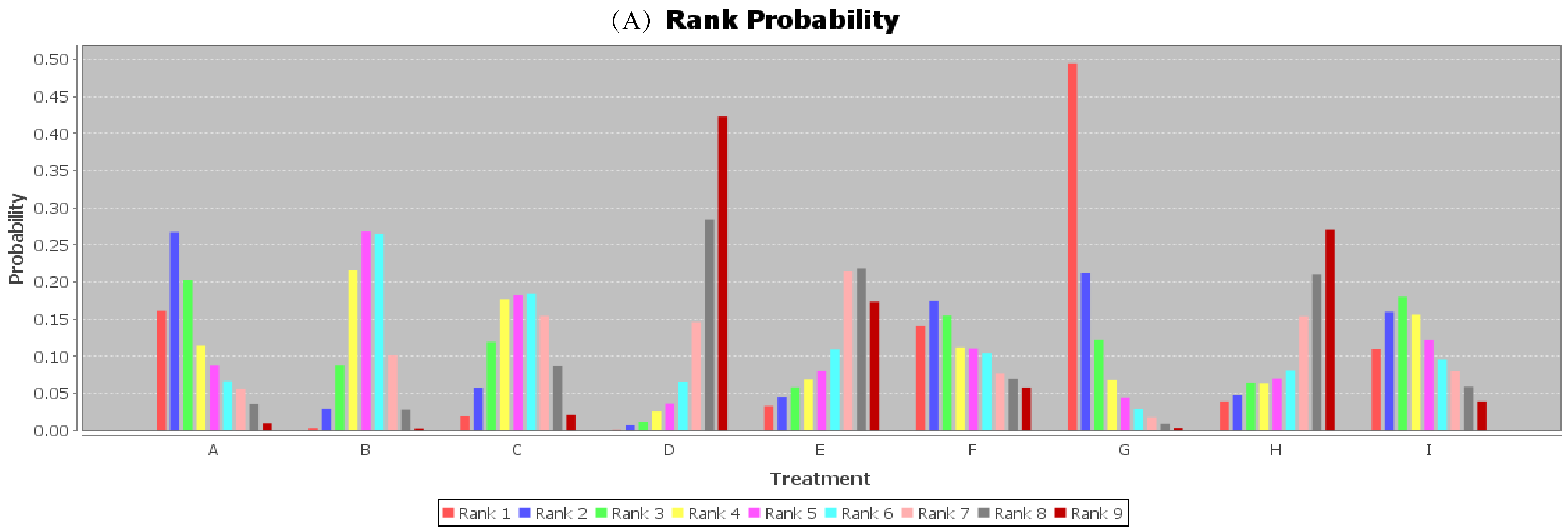

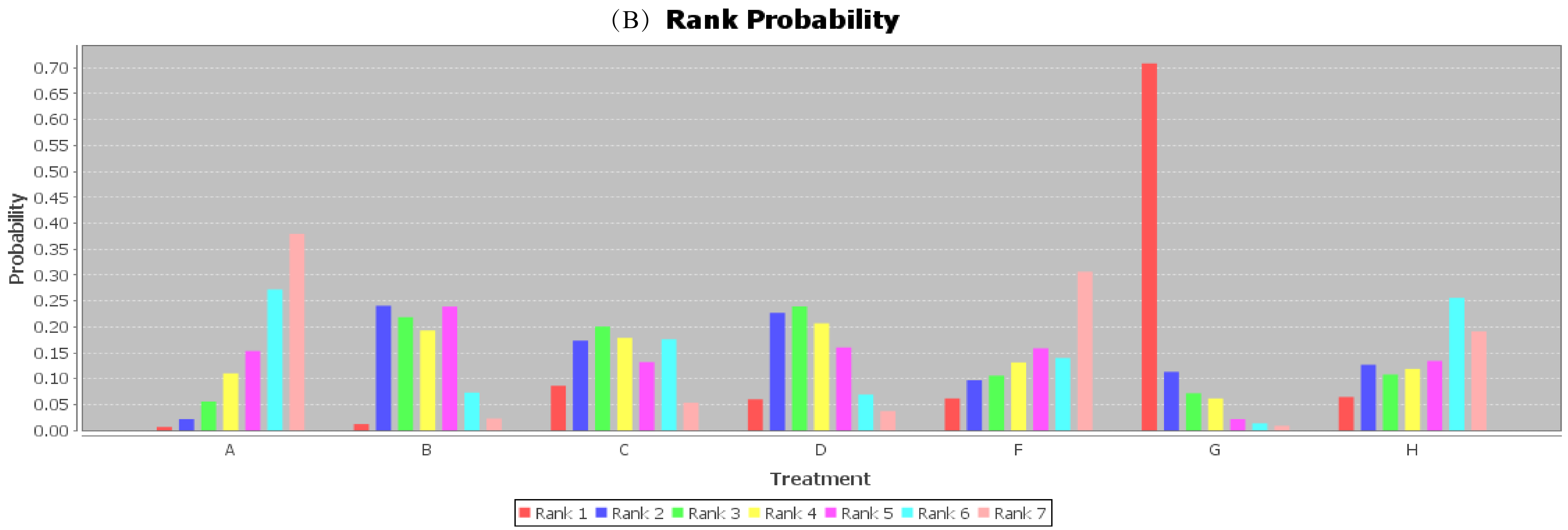

A, T-DM1; B, Lap- Cap; C, Tra-Cap; D, Cap; E, Ner; F, Per-Tra-Cap; G, Pyr-Cap; H, Ate-T-DM1; I, Ner-Cap
T-DM1, trastuzumab emtansine; Lap, lapatinib; Tra, trastuzumab; Cap, capecitabine; Ner, neratinib; Per, pertuzumab; Pyr, pyrotinib; Ate, atezolizumab
